# Composite Design of In-House Manufactured Patient-Specific Instruments for Precision Mandibular Reconstruction Using Fibula Free Flap

**DOI:** 10.7759/cureus.96073

**Published:** 2025-11-04

**Authors:** Risa Yamamoto, Yuki Wakabayashi, Midori Kawamura, Tomohiro Michida, Ken-ichiro Sakata, Masahiro Hojo, Taku Maeda, Keisuke Nakamura, Taku Kimura, Shinichiro Kuroshima, Ikuya Miyamoto

**Affiliations:** 1 Dentistry, Hokkaido University Hospital, Sapporo, JPN; 2 Oral Medicine, Oral Surgery, Hokkaido University, Sapporo, JPN; 3 Plastic Surgery, Hokkaido University Hospital, Sapporo, JPN; 4 Dentistry, Hokkaido University, Sapporo, JPN; 5 Oral Medicine, Oncolgy, Hokkaido University, Sapporo, JPN

**Keywords:** ‎3d printing, computer-assisted surgery, in-house production, mandibular reconstruction surgery, patient-specific instruments

## Abstract

Mandibular reconstruction requires precise surgical approaches, not only to achieve anatomical restoration but also to recover functional occlusion and aesthetic appearance. In our hospital, computer-assisted surgery (CAS) is used for preoperative simulation in maxillofacial reconstruction. Patient-specific instruments (PSIs) are designed with computer-aided design software and fabricated using a 3D printer. A 78-year-old male with left mandibular gingival cancer underwent fibular free flap reconstruction. Cutting, repositioning, and fibula assembly guides were designed to maintain mandibular position. Surgery was successfully performed using PSIs. Although CAS and PSIs enabled efficient reconstruction, differences between preoperative simulation and postoperative morphology were observed, indicating the need for further refinement.

## Introduction

Extensive jaw resection and reconstruction surgery are often performed to treat malignant tumors of the oral and maxillofacial region and refractory osteomyelitis of the jaw. However, even when appropriate mandibular reconstruction surgery is performed, there is still some degree of postoperative cosmetic and functional deterioration (occlusion, chewing, swallowing, and speech). Therefore, mandibular reconstruction requires precise surgical approaches, not only to achieve anatomical restoration but also to recover functional occlusion and aesthetic appearance.

Planning for fibular reconstruction surgery traditionally involved printing bone CT images at actual size on three planes and then molding a metal template to match them [1,2]. Recently, the adoption of computer-assisted surgery (CAS) has enabled accurate 3D preoperative simulation, significantly enhancing the precision and safety of reconstructive procedures [3]. Our hospital began using the virtual surgical planning software ProPlan CMF (Materialise; Leuven, BEL) around 2020 and currently employs its successor software (Enlight CMF®; Materialise) [4,5]. Its use has also been reported in several literature sources. Particularly, patient-specific instruments (PSIs), customized according to individual anatomical characteristics, have contributed substantially to standardization, efficiency of resection, reconstruction techniques, and reducing surgical time and ischemia time [4-17].

The fabrication of PSI begins with the segmentation of Digital Imaging and Communications in Medicine (DICOM) data obtained from CT. Virtual osteotomy is then performed using simulation software. The data are subsequently transferred to computer-aided design (CAD) software for PSI design, and the final model is fabricated by 3D printing with a photosensitive resin.

At our institution, CAS is utilized for preoperative simulation in reconstructive procedures involving the maxilla and mandible. Through interdisciplinary collaboration among plastic surgeons, oral surgeons, prosthodontists, and dental technicians during CAS planning, PSIs are tailored precisely to the surgical team's requirements. Based on preoperative planning, PSIs are designed by in-house dental technicians using CAD software and fabricated with a 3D printer. Conventional PSI utilized one PSI component per mandibular osteotomy site. Concerns exist that slight lateral displacement of the PSI position or significant displacement of the condyle, which is freed after mandibular osteotomy, could cause deviation from the simulated position. Therefore, measures to suppress condylar displacement after mandibular osteotomy were necessary.

Furthermore, outsourcing PSI manufacturing to systems like the TRUMATCH® CMF reconstruction system (DePuy Synthes, Raynham, MA, USA) or Cosmofix® (Cosmofix Technovation Pvt Ltd., Mumbai, MH, IND) presents disadvantages such as cost, time constraints, and limited planning flexibility. In-house PSI manufacturing offers a potential solution [10,11]. In this report, we present a case in which PSIs were fabricated in-house using CAS, with the aim of achieving rigid reconstruction while maintaining proper mandibular position. To our knowledge, this surgical technique is the first to be reported in the world.

## Case presentation

**Figure 1:**
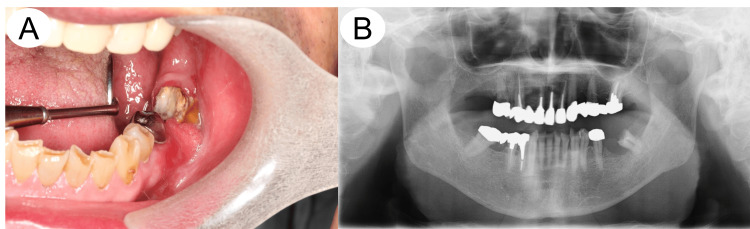
Clinical and panoramic findings at the first appearance A: Intraoral examination revealed a hemorrhagic mass measuring 20 × 15 mm in size, along with ulcer formation in the left buccal mandibular gingiva. B: Panoramic X-ray detected alveolar bone resorption with surrounding bone enhancing sclerotic changes in the left mandibular molar region.

The patient was a 78-year-old male. His medical history included hypertension, type 2 diabetes mellitus, and a gag reflex; he had no history of alcohol or smoking. Six months prior to his first visit to our department, he presented with swollen gingiva around the left mandibular molar that was initially diagnosed as periodontitis by his family dentist. Further periodontal treatment did not improve the lesion, and he was subsequently referred to our department for detailed examination. His chief complaint was spontaneous pain in the left mandibular gingiva. Extraoral examination revealed two enlarged lymph nodes without tenderness in the left submandibular region. Intraoral examination found a hemorrhagic mass measuring 20 × 15 mm accompanied by ulcer formation in the left mandibular gingiva (Figure 1A). There were no sensory abnormalities in the area controlled by the lingual and inferior alveolar nerves. A panoramic X-ray identified alveolar bone resorption with surrounding bone enhancing sclerotic changes in the lesion site (Figure 1B).

**Figure 2:**
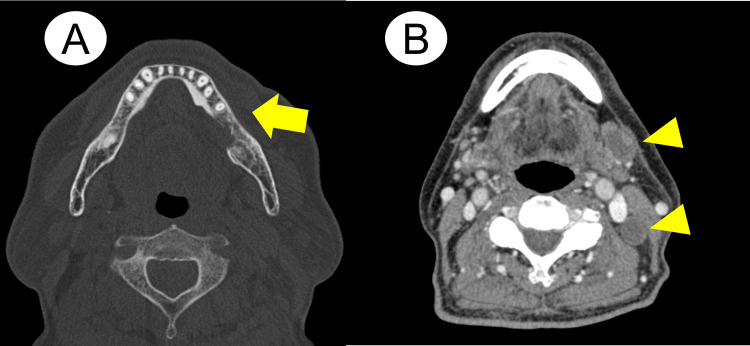
Contrast enhanced CT findings at first appearance A: Bone resorption was detected in the left mandibular alveolar region (arrow); B: Multiple cervical lymph nodes displayed rim-enhancing findings, indicating lymph node metastases (arrowheads)

**Figure 3:**
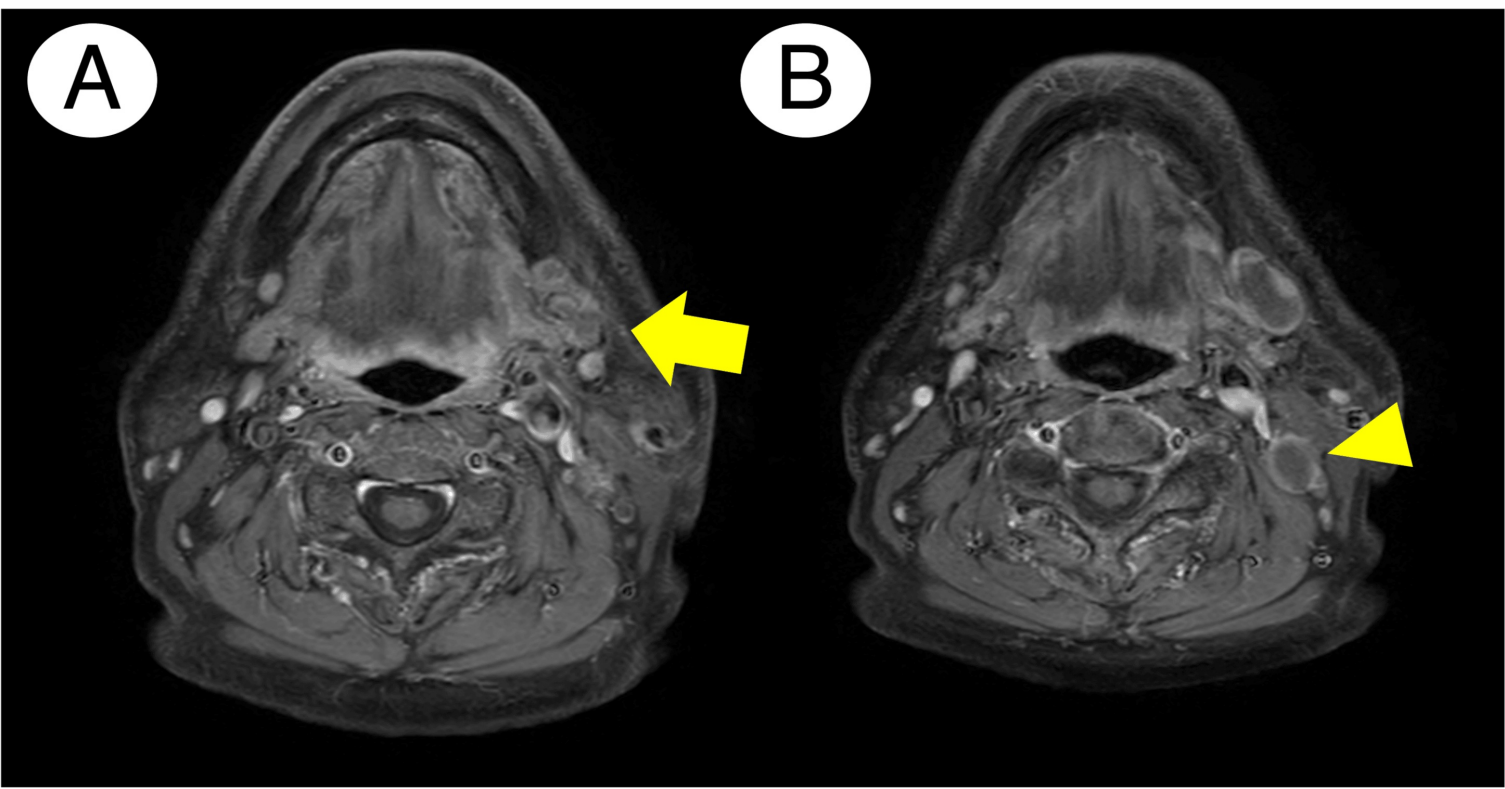
Contrast-enhanced MRI findings at the first appearance A: T1-weighted image with gadolinium contrast revealed that a heterogeneous, markedly enhancing mass lesion infiltrated into the left mandibular marrow (arrow); B: Cervical lymph node displayed rim-enhancement findings, indicating lymph node metastasis (arrowhead shows a representative metastatic lymph node)

Contrast-enhanced CT depicted a mandibular invasion finding (Figure 2A). A contrast-enhanced MRI demonstrated a mass lesion extending into the left mandibular marrow without apparent infiltration into the masticator space (Figure 3A). Additionally, both contrast-enhanced CT and MRI showed multiple left cervical lymph nodes with a rim-enhancement pattern (Figures 2B-3B).

Ultrasound examination also identified a sign of lymph node metastases in the left cervical region. Accordingly, a clinical diagnosis left a mandibular gingival tumor suspicious of squamous cell carcinoma (SCC) with multiple cervical lymph node metastases. A scraping cytology identified signs of SCC, including cellular atypia, cytoplasmic changes, and irregular cell shapes. Therefore, the patient's preoperative diagnosis was left mandibular gingival SCC with lymph node metastases (cT4aN2bM0, stage ⅣA). 

Normally, a biopsy would be necessary, but he had a gag reflex and was unable to undergo a biopsy. We only performed a cytology test before the surgery. After evaluation by the clinical team, the patient underwent segmental mandibular resection and reconstruction with a fibula free flap, along with tracheostomy and modified radical neck dissection (type III, levels I-Ⅴ). The operation duration was 12 hours and 40 minutes, with an intraoperative blood loss of 930 mL. The CT findings were bone destruction up to the inferior alveolar nerve; a segmental mandibular resection was selected. Pathological examination revealed SCC with 11 left cervical lymph node metastases (11/37 in total, including 0/1 in level A; 2/2 in level ⅠB; 4/5 in level ⅡA with extranodal extension; 3/11 in level Ⅲ; 1/13 in level Ⅳ; 0/4 in level ⅤA; and 1/1 in level ⅤB). Consequently, the patient was diagnosed with left gingival SCC with lymph node metastases, pT4aN3bM0 (stage ⅣB), and underwent postoperative radiotherapy with 60 Gy in 30 fractions in total.

**Figure 4:**
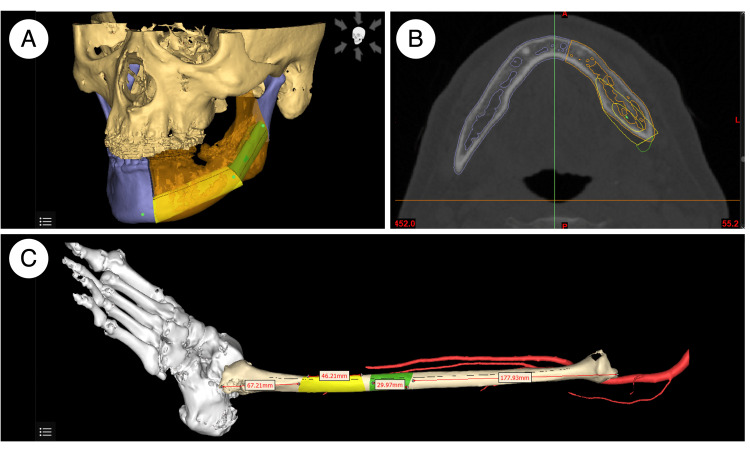
Reconstruction planning using dedicated 3D planning software A: Fibular flap design (3D model); B: Fibular flap design (CT horizontal plane); C: Fibular design

As for the preparation of the PSI, preoperative CT scans of the face and lower extremities (0.5 mm slice thickness) were obtained, and 3D anatomical models of the mandible and fibula were generated from DICOM data using 3D segmentation software (medical image processing software) (Mimics; Materialise). Reconstruction planning was conducted using dedicated 3D planning software (Enlight CMF®; Materialise). The surgical plan was finalized through multidisciplinary conferences involving plastic surgeons, oral surgeons, prosthodontists, and dental technicians. Subsequently, several PSIs were designed using CAD software (3-matic®; Materialise) (Figure 4).

**Figure 5:**
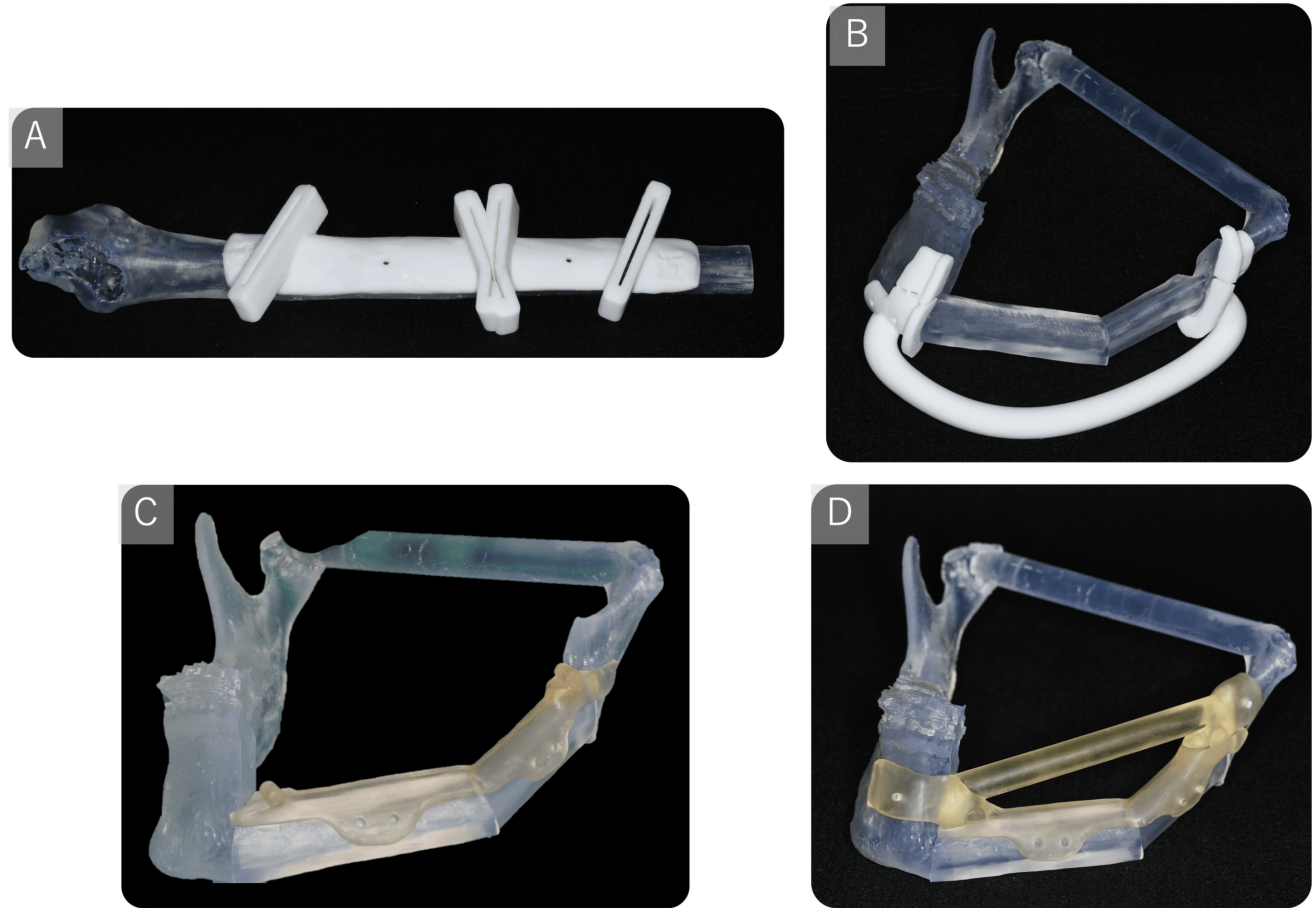
The various PSIs A: Fibula cutting guide; B: Mandibular cutting guide; C: Fibula assembly guide; D: Positioning guide plus lower part of the mandibular cutting guide PSIs: Patient-specific instruments

**Figure 6:**
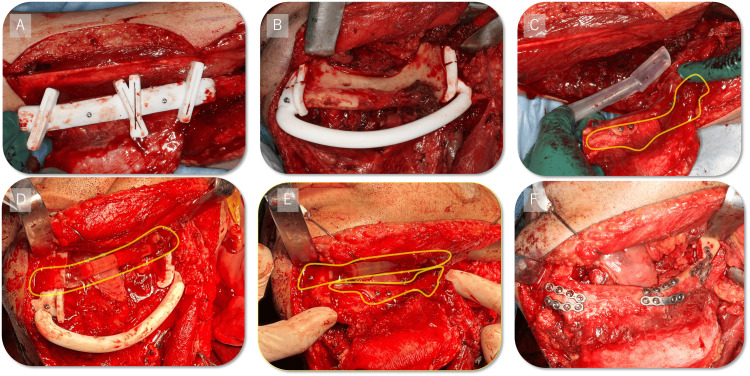
Intraoperative photographs A: Placement of fibula cutting guide; B: Placement of mandibular cutting guide; C: Fibula assembly guide; D: Positioning guide plus fibula assembly guide; E: Positioning guide plus lower part of the mandibular cutting guide; F: Fixation of the reconstruction plate

The fibular cutting guide was precisely designed to facilitate accurate osteotomies at predetermined angles and lengths according to preoperative simulations (Figure 5A and Figure 6A). The mandibular cutting guide enabled precise osteotomy along resection margins and incorporated a split mechanism allowing separation into upper and lower components (Figure 5B and Figure 6B). Additionally, a fibular assembly guide was fabricated to preassemble fibular segments (Figure 6C), ensuring optimal alignment during mandibular reconstruction (Figure 4C). Furthermore, a positioning guide was developed to maintain condylar position intraoperatively by securing both ends of the mandibular osteotomy site (Figure 5D and Figure 6D). After removal of the lower component of the mandibular cutting guide, the fibular assembly guide and positioning guide were integrated (Figure 6E) and stabilized with mini-plates (Figure 6F), enabling mandibular reconstruction without compromising continuity.

The 3D printing was performed using a Form 3D printer (Formlabs Inc., Somerville, MA, USA) at a 0.1 mm layer thickness, utilizing BioMed white and surgical guide resin to achieve the required intraoperative mechanical strength and dimensional accuracy. Postoperative CT scans obtained one month after surgery were aligned with the preoperative simulation stereolithographic data based on the residual mandibular segments to assess condylar positioning. Postoperatively, our patient did not experience major complications, including apparent occlusal changes or impairment of oral functions. A CT scan one month later showed no recurrence in either the primary lesion or metastatic lymph nodes. Following the procedure, occlusal tapping was performed as part of rehabilitation. He was discharged from our department one month after the surgery. He is now under regular monitoring every three months, without exhibiting a recurrence.

The integration of multiple PSIs facilitated a smooth and efficient surgical procedure. Notably, the positioning guide enabled precise intraoperative maintenance of condylar position, thus ensuring accurate placement and fixation of the reconstruction plate. The CT imaging conducted one month postoperatively confirmed stable condylar positioning and favorable soft-tissue contours. Intraoperative photographs showed stepwise application of PSIs culminating in reconstruction plate fixation (Figure 6, panels A to F).

**Figure 7:**
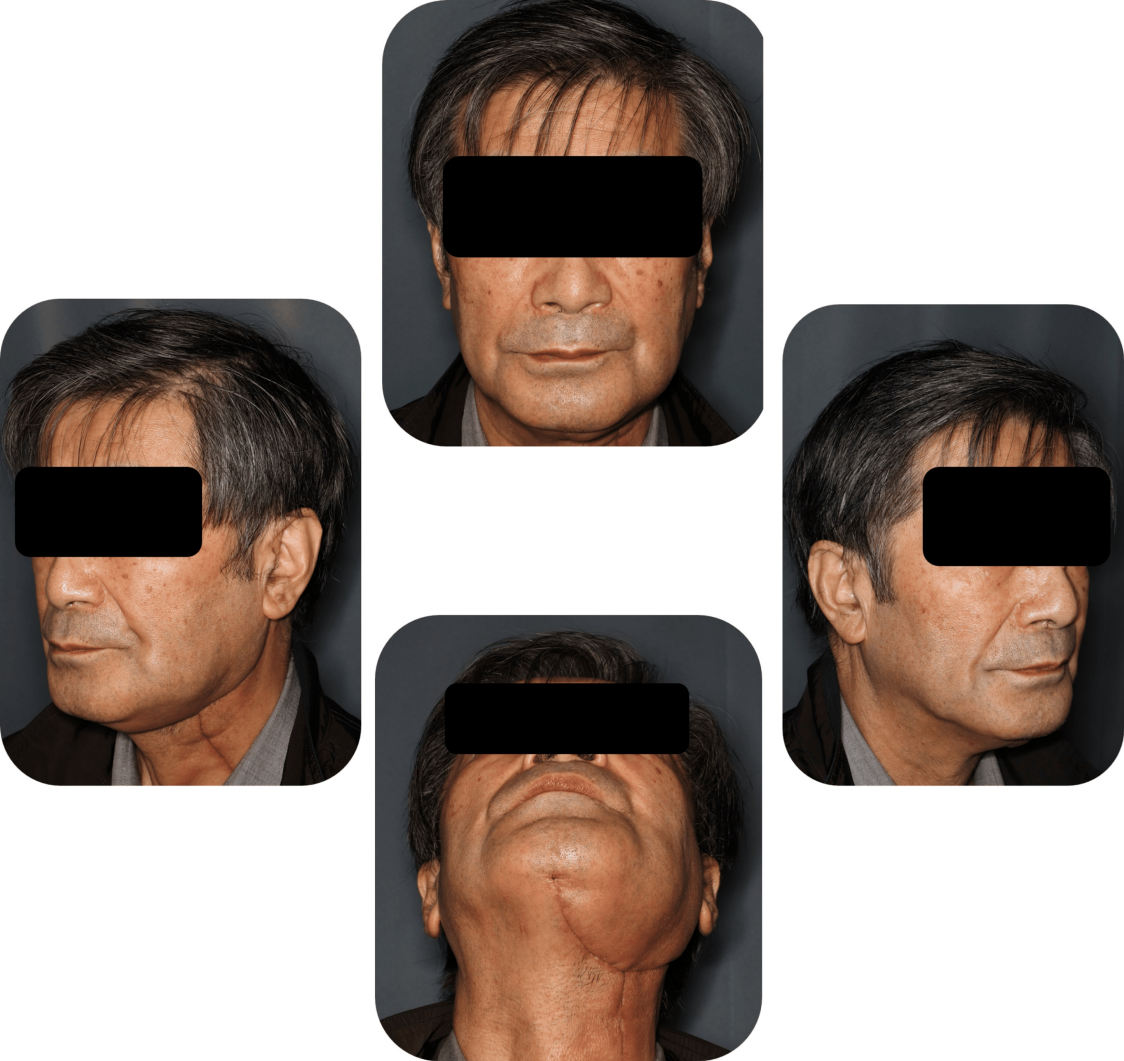
Facial appearance one month postoperatively

**Figure 8:**
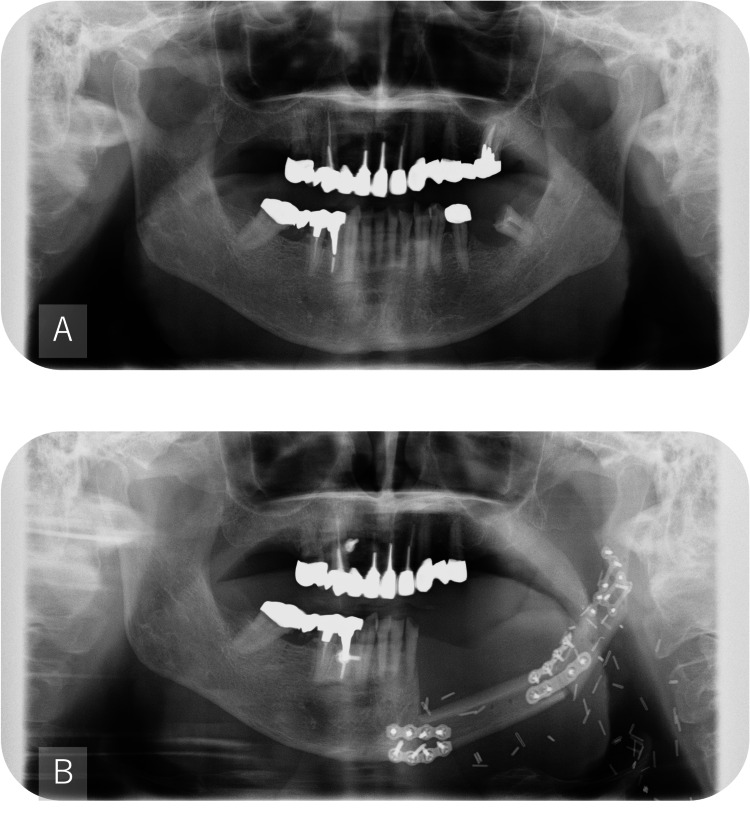
Panoramic radiographs A: Preoperative; B: One month postoperative

**Figure 9:**
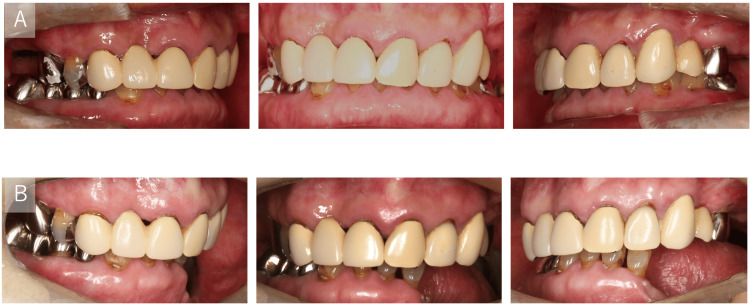
Intraoral photographs A: Preoperative; B: 10 months postoperative

**Figure 10:**
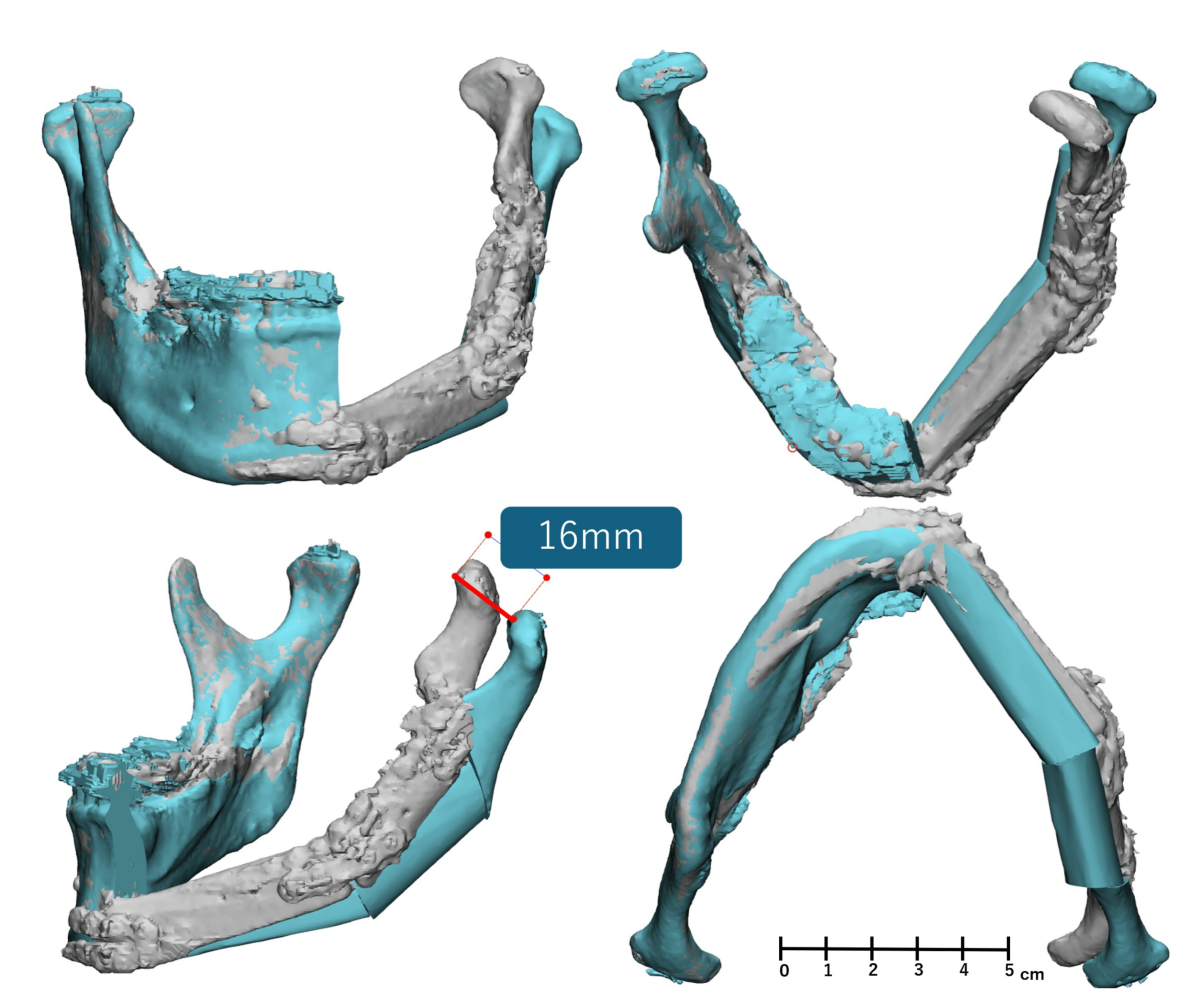
Superimposition of preoperative simulation and postoperative STL data Blue: preoperative planning; White: One month postoperative Imaging method: CT (slice thickness 0.5 mm); Osteotomy angle: 90°relative to the mandible STL: Stereolithography

Extraoral examination at one month showed a symmetric facial contour without conspicuous swelling (Figure 7). Postoperative panoramic radiograph at one month confirmed stable reconstruction plate fixation with restored mandibular continuity (Figure 8, panel B). Intraoral examination at 10 months demonstrated healed mucosa and stable occlusion (Figure 9, panel B). The position of the left mandibular condyle deviated 16 mm toward the affected side compared to its position on the preoperative CT image (Figure 10). However, even though the position of the mandibular condyle changed after the surgery, with a maximum morphological deviation of 16 mm toward the affected side, no occlusion abnormalities occurred (Figure 9B).

## Discussion

This case demonstrates that the integration of multiple PSIs can significantly enhance the accuracy and reproducibility of mandibular reconstruction procedures. Particularly, the introduction of a positioning guide effectively stabilized the placement of the reconstruction plate and preserved the condylar position intraoperatively, representing a novel approach highlighted by this case. Beyond this case, prior reviews indicate that computer-assisted surgery (CAS), spanning virtual planning, navigation, and CAD/computer-aided manufacturing (CAM)-based PSIs, improves geometric accuracy and can increase time efficiency in oral-maxillofacial reconstruction [18,19]. The PSIs help translate the virtual plan intraoperatively, reduce manual plate bending, and enhance fit/contour; CAS/PSI workflows have also been associated with shorter operative and ischemia times [18,19]. Furthermore, the accuracy of PSI is guaranteed to be within the acceptable range for surgical cases [20-23], making it beneficial for mandibular reconstruction cases requiring precise reconstruction, such as occlusion. However, evidence comparisons remain limited by heterogeneity in planning and evaluation methods, which constrains meta-analysis and direct cross-study benchmarking [3]. Practical drawbacks of CAS/PSI workflows include software/hardware cost, workflow complexity and training needs, difficulty in objectively assessing soft-tissue accuracy, and the possibility that rigid plans or misuse can prolong operative time or hinder intraoperative adaptation [19,24].

**Figure 11:**
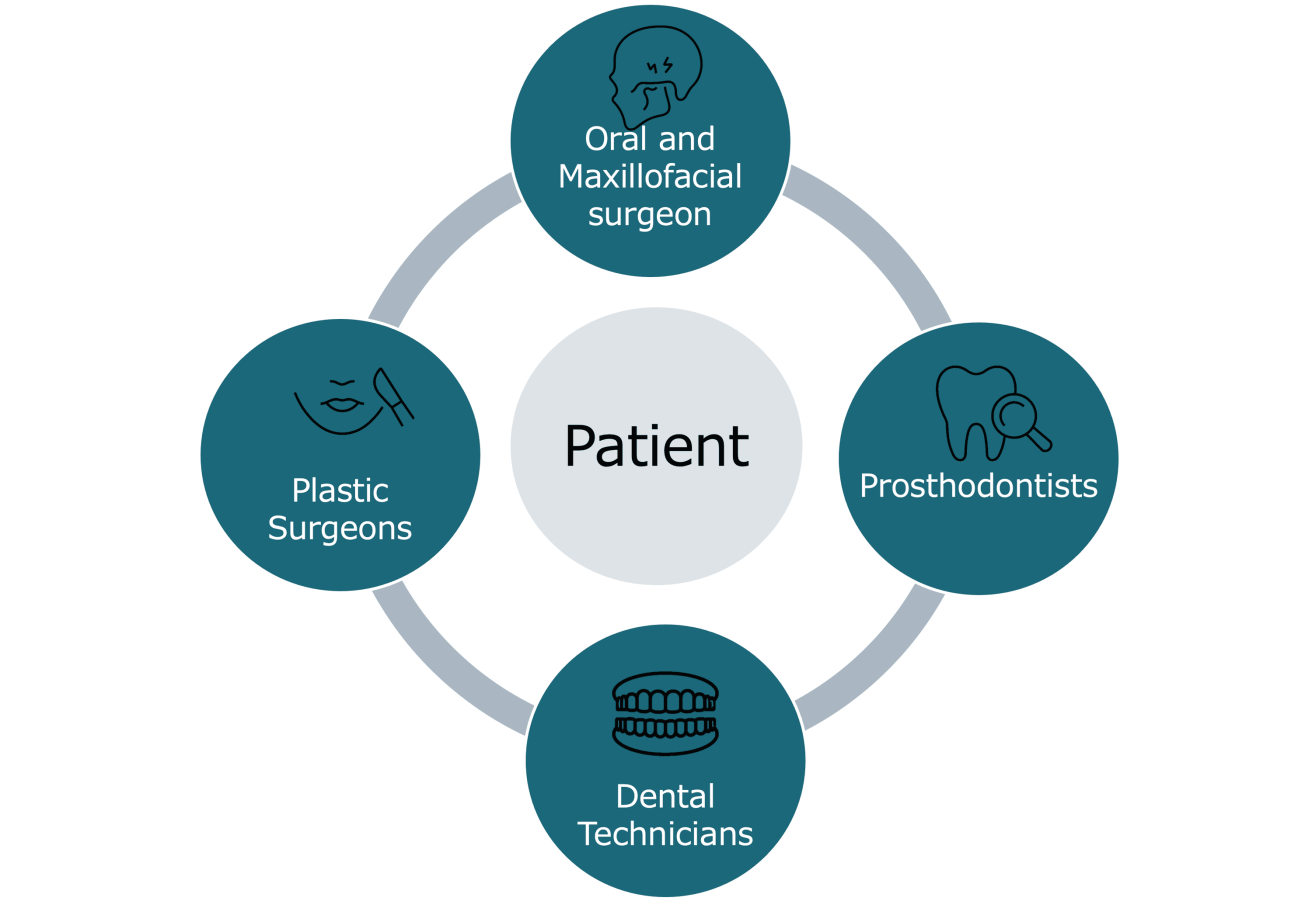
Patient-centered multidisciplinary collaboration in the in-house fabrication of PSIs A multidisciplinary team approach is important. PSIs: Patient-specific instruments Illustration is a royalty-free image obtained from Adobe Stock.

Critical considerations include precise preoperative plate bending, postoperative occlusal stabilization, and standardized CT imaging conditions. Although the combined use of cutting guides and patient-specific plates has become routine in orthognathic surgery [25], such an integrated approach remains uncommon in malignant tumor cases [16]. The present case, employing multiple PSIs (cutting, assembly, and positioning guides), successfully maintained mandibular positioning while achieving high mandibular reconstruction accuracy, underscoring its clinical significance. Regarding the cost of PSI fabrication, outsourcing custom-made plates such as the TRUMATCH® CMF reconstruction system can require up to one month for delivery, while domestically produced, fully customized plates like Cosmofix® take approximately two weeks [15]. In contrast, in-house PSI fabrication can be completed in less than two weeks, reducing outsourcing costs [4]. Moreover, in-house production facilitates close communication between dental technicians responsible for PSI design and the surgical team, including plastic surgeons, oral and maxillofacial surgeons, and prosthodontists, thus promoting interdisciplinary collaboration centered on the patient (Figure 11).

Nevertheless, the morphological deviation observed from the preoperative plan highlights several areas for potential improvement. Contributing factors for postoperative deviation may include intraoperative misalignment of the reconstruction plate, biomechanical changes resulting from muscle resection, variations in screw insertion angles, and positional discrepancies during CT imaging. Similar postoperative deviations have been reported following mandibular sagittal split ramus osteotomy (SSRO), suggesting that dynamic mechanical influences, altered occlusal forces, and changes in muscular tension unique to the maxillofacial region may have played significant roles [26,27]. In this case as well, despite the use of PSI designed to maintain the planned mandibular position, a maximum deviation of 16 mm was observed. This discrepancy may be attributed to changes in occlusal forces during and after surgery, alterations in masticatory muscle tension, and dynamic mechanical factors unique to the maxillofacial region. To address these, postoperative intermaxillary fixation is necessary. Additionally, previous studies have shown that in orthognathic surgery, particularly SSRO, the degree of condylar displacement increases when the glenoid fossa is shallow [28]. Given that the average depth of the glenoid fossa is reported as 8.15 ± 0.51 mm, the relatively shallow depth of 5.15 mm observed in this patient may have contributed to the observed displacement. Moreover, discrepancies between virtual surgical planning and actual postoperative outcomes have been documented in prior studies [29,30], emphasizing the necessity to incorporate biomechanical and operational variables into reconstruction planning. Furthermore, providing psychological motivation to patients leads to better outcomes. Future efforts should focus on optimizing intraoperative and postoperative intermaxillary fixation methods, enhancing the precision of plate bending, and accumulating clinical experience to develop consistently reproducible reconstructive techniques.

## Conclusions

In this case, using multiple PSIs enabled precise mandibular fibula reconstruction based on preoperative simulation to successfully maintain mandibular positioning and provide functional and aesthetic outcomes. Furthermore, in-house fabrication of the guides reduced both the time and cost compared to outsourced production. However, differences between preoperative simulation and postoperative morphology were observed, indicating the need for further refinement.
